# Reasons for presenting to antenatal care clinics in a sample of Pakistani women and their knowledge of WHO antenatal care package

**DOI:** 10.18332/ejm/140794

**Published:** 2021-10-01

**Authors:** Syed H. Ala, Samia Husain, Saba Husain

**Affiliations:** 1 Department of Obstetrics and Gynecology, Dow Medical College, Dow University of Health Sciences, Karachi, Pakistan; 2 Department of Obstetrics and Gynecology, Aziz Medical Center, Karachi, Pakistan

**Keywords:** reasons, antenatal care, World Health Organization (WHO) antenatal care package, low-middle income countries (LMIC)

## Abstract

**INTRODUCTION:**

The purpose of antenatal care is to ensure that a woman has a safe pregnancy and that does not mean absence of any disease during this period. Antenatal care allows screening of preeclampsia, fetal abnormalities and other prevention strategies to be incorporated. The purpose of this study was to assess the reason for attending antenatal care clinics and knowledge of antenatal care content package in women.

**METHODS:**

A cross-sectional study was conducted on 395 pregnant women attending antenatal care clinic at the Ruth K. M. Pfau Civil hospital, Karachi, Pakistan from 1 July 2019 to 31 December 2019. Each eligible woman was asked about the reason for attendance and her knowledge about WHO standardized antenatal care package.

**RESULTS:**

The commonest reason for utilizing antenatal care in booked attendees was place of birth concern (25.9%) and in not booked was referral from private centers (33.6%) which was statistically significant (p=0.006). Both booked and not booked women (67.9% vs 59.1%, p=0.409) stated avoidance of complication during pregnancy and labor as the commonest reason for attendance. Women with higher parity were more likely to identify weight measurement (p=0.001), iron and folic acid supplementation (p=0.001), and urine detailed report (p=0.002), as content of the standard package.

**CONCLUSIONS:**

Our study shows that women did not utilize antenatal care clinics for improving their health or the health of their fetus. The knowledge of the antenatal care package was limited to weight measurement and supplements. Moreover, attendance and visits at an antenatal care facility do not equate to good service provision.

## INTRODUCTION

Antenatal care is not just a regular health check for pregnant women but serves a unique purpose. The purpose of antenatal care is to ensure that a woman has a safe pregnancy and that does not mean absence of any disease during this period^1^. Antenatal care allows screening of preeclampsia, fetal abnormalities and other prevention strategies to be incorporated^2^. But the utilization of antenatal care by women in low- and middle-income countries is very different from that of high-income countries. Although the utilization has increased considerably in the past two decades, the reasons for utilization are not very convincing^3^.

A visit should ideally incorporate the pregnancy specific care but the common reasons for accessing an antenatal care facility by Pakistani women were surprisingly different^4^. In a multicenter study, the Pakistani women were more concerned with birth preparation, and were less inclined to utilize any other preventive or screening services offered at the facility^5^.

The antenatal services are not utilized to their full potential in the country. It has been reported that women seldom present for antenatal care or present really late^6^. The birth specific complaints or pregnancy related concerns form a minor fraction of presenting complaints^7^.

Presentation to the antenatal care clinic is often the only time a woman comes in contact with the health professional. But this increasing attendance at antenatal care in Pakistan can merely pass for better service provision and not actual service utilization. There is a need to motivate women not to just access this service but utilize it to its true potential. Antenatal care is the best platform for screening, educating and providing needed healthcare during pregnancy. We conducted this study to assess the reasons for utilizing antenatal care in women presenting to an outpatient department.

## METHODS

The study was conducted from 1 June 2019 to 31 December 2019, and was carried out in the outpatient clinic of obstetrics and gynecology of the Ruth K. M. Pfau Civil Hospital, Karachi. Karachi is the biggest city of Pakistan and has a population of about 20 million^8^. The specific civil hospital is the largest tertiary care public sector hospital that receives patients from all walks of life. The delivery rate at this civil hospital is 7500 deliveries per year, and around 200 women come for antenatal care each day. The antenatal clinic is held daily from Mondays through Saturdays, from 9 a.m. to 1 p.m. These clinics are run by consultant obstetricians with their teams of resident doctors and are assisted by nurses.

### Inclusion and exclusion criteria

All pregnant women aged 20–40 years attending antenatal clinic were informed and invited. Brief history and informed consent were obtained from each patient. Women in active labor on history and examination, women who had active bleeding or needed emergency treatment were excluded. Women who refused to consent were also excluded. A woman was considered to have adequate ANC visits and inadequate visits according to old WHO criteria of 2014^9^.

Women who met the inclusion criteria were asked about antenatal care and the reasons for which they presented to the antenatal clinic. The findings of variables were entered in pre-designed proforma attached as an annex.

### Power of sample size

To calculate an adequate sample size we searched the literature for reasons for utilizing antenatal care. There is only one study which directly quoted reasons for antenatal care utilization^10^. Using that study as reference and assuming similar proportion for this population, the sample size for the study was determined as n=359. The sample size was calculated using the WHO software where, true utilization of antenatal care for pregnancy was 37%, margin of error=5% and 95% confidence level. We used the estimating population proportion with specified absolute precision test to calculate the sample size (Sample Size Determination in Health Studies, Version 2.00, Copyright (c) 1996–98, World Health Organization). To compensate for incomplete responses, the sample size was increased by 7% so that 384 women were included.

### Statistical analysis

Data was analyzed using SPSS Version 16. Means and standard deviations were calculated for the quantitative variables, such as maternal age and gestational week at which a woman presented. Frequencies and percentages were calculated for the qualitative variables, such as monthly income in PKR (low: ≤10000, middle 10000– 40000, and high >40000), occupational status, educational level, and parity. The classification for monthly income was as previously used in local studies^11^. Effect modifiers were controlled through stratification of maternal age, monthly income, occupational status, educational level and parity, to see the effect of these on the outcome variable. Post-stratification chi-squared test was applied taking p≤0.05 as statistically significant.

A bivariate analysis was done to assess the association of reasons for antenatal care utilization with adequate antenatal visits. The Pearson chi-squared test was used to assess associations between variables for an alpha error of 5%.

A further analysis was conducted to assess the knowledge of these women with respect to WHO package for minimum criteria. Each woman was asked about the seven essential variables: weight measurement, blood pressure measurement, blood tests, tetanus toxoid, urine detailed report, iron/folic acid, and counselling for dangerous signs. Women were stratified according to their parity: primigravida, parity ≤3, and >3. The parity classification was used because the total fertility rate for Pakistani women is 3.212. As parity increases the exposure of women to antenatal service is expected to increase. This association of parity with knowledge of the care package was assessed by a separate analysis.

## RESULTS

### Basic demographic and clinical characteristics of studied population

During the study period 384 women were approached. Of these, 16 women refused to consent and 9 were excluded due to incomplete responses. We therefore included 359 women. Mean age of the participants was 31.12 years. Of these women, 47.6% had 3 or less children and had 2–4 visits (42.6%). Only 31.2% had adequate antenatal care and the majority (87.5%) had presented for the first time after 14 weeks of gestation.

Among the attendees, 171 (41.6%) had primary education, 185 (51.5%) were of low socioeconomic status (51.5%), 80.5% resided in an urban area and 53.5% were unemployed.

Of those who presented at the clinic, 206 (57.4%) had previously delivered vaginally and only 13.9% were having their first baby. Most of these women (192; 53.5%) had delivered at other government hospitals, while only 16.7% of the women had previously delivered at the Civil Hospital, Karachi. The characteristics of the population studied are summarized in Table 1.

**Table 1:** Characteristics of study population (N=359)

*Characteristics*	*Categories*	*n (%)*
**Age** (years), mean±SD		31.2±2.1
**Parity**	>3	138 (38.4)
	≤3	171 (47.6)
	Primigravida	50 (13.9)
**Number of visits**	0–1	94 (26.2)
	2–4	153 (42.6)
	<4	112 (31.2)
**Adequate antenatal visits** (>4 visits)	Yes	112 (31.2)
	No	247 (68.8)
**Previous mode of delivery**	First baby (primigravida)	50 (13.9)
	Vaginal birth	206 (57.4)
	Cesarean delivery	103 (28.7)
**Previous place of delivery**	First baby (primigravida)	50 (13.9)
	Civil hospital	60 (16.7)
	Other government hospitals	192 (53.5)
	Private hospitals	57 (15.9)
**Place same**	First	50 (13.9)
	Yes	60 (16.7)
	No	249 (69.4)
**Gestational age at presentation** (weeks)	<14	45 (12.5)
	≥14	314 (87.5)
**Education level**	No formal	129 (35.9)
	Primary	171 (47.6)
	Secondary	42 (11.7)
	Graduate	17 (4.7)
**Monthly income**	Low	185 (51.5)
	Middle	150 (41.8)
	Upper	24 (6.7)
**Occupational status**	Employed	167 (46.5)
	Unemployed	192 (53.5)
**Occupation status of husband**	Employed	220 (61.2)
	Unemployed	139 (38.8)
**Residence**	Urban	289 (80.5)
	Rural	70 (19.5)

### Reasons for antenatal care utilization

The commonest reason for utilizing antenatal care in booked attendees was place of birth concern (25.9%) and in not booked was referral from private centers (33.6%), which was statistically significant (p=0.006). Both booked and not booked women (67.9% vs 59.1%, p=0.409) stated avoidance of complications during pregnancy and labor as the commonest reason for seeking antenatal care. Women however also stated that pregnancy and labor are natural processes and therefore antenatal care may not be necessary. Table 2 shows the reasons for utilization and factors identified by women in favor of and against utilization of the care provided.

**Table 2:** Reason for utilization in women with adequate antenatal visits and inadequate visits

	*Adequate (n=112) n (%)*	*Inadequate (n=247) n (%)*	*p*
**Reason for utilization**			0.006*
Pregnancy specific reason	11 (9.8)	9 (3.6)	
Baby related reason	11 (9.8)	10 (4.0)	
Medical reason	14 (12.5)	22 (8.9)	
Screening for anomaly	2 (1.8)	19 (7.7)	
Previous section	12 (10.7)	24 (9.7)	
Place of birth concern	29 (25.9)	67 (27.1)	
Referral from private	23 (20.5)	83 (33.6)	
Medicine and free labs	4 (3.6)	4 (1.6)	
Financial	6 (4.5)	9 (3.6)	
**Factors identified by women attending for ANC in favor of utilization**			0.409
To avoid complication during pregnancy and labor	76 (67.9)	146 (59.1)	
Affordability	27 (24.1)	70 (28.3)	
Health facilities are up to the mark	5 (4.5)	18 (7.3)	
Doctors are good	4 (3.6)	13 (5.3)	
Family issue	4 (3.6)	20 (8.1)	
**Factor identified by women attending for ANC against utilization**			0.140
Financial	25 (22.3)	71 (28.7)	
Transport	12 (10.7)	27 (10.9)	
Pregnancy and labor are natural processes so no need to come	55 (49.1)	109 (44.1)	
They can check me at home	16 (14.3)	20 (8.1)	

* Chi-squared test or Fischer’s exact test is significant at p<0.05.

### Knowledge of criteria and association with parity

Table 3 shows stratification of women according to parity and the parameters they identified according to WHO criteria for antenatal care. Women with higher parity were more likely to identify weight measurement (p=0.001), iron and folic acid supplementation (p=0.001), and urine detailed report (p=0.002), as content of the standard package. However, the identification did not vary significantly in terms of counselling for danger signs (p=0.586), blood pressure (p=0.056), blood count (p=0.067), and tetanus toxoid administration (p=0.282).

**Table 3:** World Health Organization’s standardized antenatal care package content identified by women attending antenatal care clinic

*Component*	*Primigravida (n=50) n (%)*	*Parity ≤3 (n=171) n (%)*	*Parity >3 (n=138) n (%)*	p*
Weight measurement	44 (88.0)	110 (64.3)	123 (89.1)	0.001*
BP measurement	48 (96.0)	142 (83.0)	121 (87.7)	0.056
Blood tests	35 (70.0)	120 (70.2)	112 (81.2)	0.067
Tetanus toxoid	10 (20.0)	51 (29.8)	33 (23.9)	0.282
Urine detailed report	7 (14.0)	83 (48.5)	74 (53.6)	0.002*
Iron/folic acid	26 (52.0)	136 (79.5)	109 (79.0)	0.001*
Counselling for dangerous signs	5 (10.0)	27 (15.8)	21 (15.2)	0.586

* Chi-squared test or Fischer’s exact test is significant at p<0.05.

## DISCUSSION

Our study shows that antenatal care perception in women was more of a facility-based birth. They did not utilize it for improving their health or that of their fetus. The knowledge of antenatal care package was limited to weight measurement and supplements. The parity also did not affect this knowledge in terms of blood pressure measurement, blood count, tetanus toxoid administration, and counselling for danger signs in pregnancy.

Prenatal and antenatal care are the cornerstone of modern obstetrics^13,14^. The situation in Pakistan’s largest metropolitan city is alarming. Women continue to receive <4 visits which is lower than the WHO Antenatal Care Trial (WHOACT) recommended minimum. In 2001 the trial concluded that an antenatal care package of evidencebased screening, therapeutic interventions and education across four antenatal visits for low-risk women reduces cost and is not inferior to standard antenatal care^15^. Socially disadvantaged women with low household wealth are shown to suffer from lack of care during pregnancy^16^. This is in stark contrast to our study where only 31.2% of women had ≥4 visits, despite the fact that the hospital does not charge a fee for antenatal checkups. Early initiation of care ensures a better outcome for pregnant women. Women who begin care early reap more benefits from these checkups.

Studies have previously concluded that antenatal care seeking behavior is determined primarily by socioeconomic factors^17,18^. Affordability of care is of prime importance and expensive care may not be accessible to all women. However, our study was based in a public sector hospital where all antenatal care is provided free of cost, therefore this reason loses its validity and opens up a discussion on consumer attitudes. A study from Kenya^19^ found that women were dependent on their spouse and had to bear the cost of travel to reach the antenatal care clinic. Most of our respondents were unaccompanied and had travelled smaller distances and only 10% stated that travel may be a hindrance in accessing antenatal care.

A recent qualitative analysis from Cameroon concluded that most pregnant women do not place a high value on early initiation of antenatal care because they consider pregnancy a normal health condition that does not need medical care^20^ In our study, a similar theme was apparent. Their desire to access antenatal care at a facility was primarily driven by concerns about place of birth. When inquired about their reason for a visit, concerns for their health or their unborn child were secondary. Of the population that had adequate antenatal care utilization, only 25.9% stated that they come for visits to ensure that they deliver at the health facility. This negligence or lack of knowledge of the antenatal care package in the population is a hindrance to achieving sustainable development goals (SDG)^21^.

Hospital deliveries do not ensure good outcomes for mother and the neonate if the service is not utilized properly^22^ . Apparently, women have reduced antenatal care as they are only interested in getting registered at a facility where they can give birth. Access to antenatal care has markedly improved but the divide between provider and consumer has not yet narrowed. This is also a point that needs further evaluation through large surveys.

Content of care provided has been a topic of debate for some time in the medical literature. WHO has standardized the package of antenatal care^23^ . Our study also showed that the women did not receive the complete content at antenatal visit; this proves that the content of care received and access to antenatal care services are not synonymous. Primigravida women were more likely not to identify the contents of antenatal care provided. The major concern however was the low percentage of previously delivered women who failed to identify counselling for the danger signs in pregnancy as a component of the antenatal care content package. This patchy and inconsistent content delivery may also be the cause for the inadequate utilization of antenatal care service^24^.

Iron and folic acid supplementation was a common identified theme in parous females. This may be considered a positive aspect of the study, but high prevalence of anemia in the country warrants better utilization in this regard too. Primigravida women become iron deficient and the vicious cycle continues in future pregnancies. Prevention is better than cure and this problem too can be tackled if women have proper antenatal care utilization^25^.

Our study shows that attendance and visits at an antenatal care facility do not equate to good service provision. Awareness campaigns are a need of the hour. Service cannot be delivered properly if the consumer is not aware of its benefits and content.

Current world trends are favoring group antenatal care^26^. But that may be too liberal in the Pakistani context at the moment. Considering the fact that Pakistan is a developing country, much remains to be desired of the antenatal care services and service providers. In addition to that, indepth qualitative studies are necessary to understand why antenatal care is not sought in its true extent.

### Strengths and limitations

The strength of the study is its unique subject: reasons for coming to an antenatal facility. Many studies have been done on the advantages of adequate antenatal visits^13^. Some of these studies have also highlighted the hindrances that pregnant women face during their visits^14^. However, not many studies have reported reasons for attendance^10^. Our study assessed the consumer’s reason for attending antenatal care. In addition, we used the same opportunity to ask about the reason for attendance and her knowledge about WHO standardized antenatal care package. The major limitation is its single center design. The generalizability of results may therefore be a concern. But we would argue that the data were collected from the city’s largest public hospital that caters for a wide variety of ethnic groups and people from many walks of life in the city. The average attendee may therefore be representative of the whole population. We suggest further multicenter studies to confirm our findings in this regard.

The age range of the participants in the study was 20– 40 years; while it is well known that most of the prenatal complications such as preterm labor, abortion, prenatal hemorrhage etc. occur at early ages (<20 years), the Pakistani law does not allow marriage to be registered for women aged <18 years^27^. Our hospital is a public sector hospital and does not provide care for women who are underage. But, considering the high rate of early marriages in Pakistan among girls, the results may not reflect the true situation among this vulnerable population. In addition, previous studies have shown that the pregnancy complications could increase at older ages, particularly after the age of 40 years^28^. In Pakistan, women get married at an early age and complete their families mostly by mid-thirties; there were not enough women to be included in that age group^29^.

The WHO guideline (2016) has updated the adequate number of antenatal visits to 8^30^. Based on the new definition and our results, many Pakistani women do not receive an adequate number of antenatal visits during their pregnancy. This should be taken into consideration when analyzing results and devising policies for the future.

## CONCLUSIONS

Our study shows that women did not utilize antenatal care clinics for improving their health or the health of their fetus. The knowledge of the antenatal care package was limited to weight measurement and supplements. Moreover, attendance and visits at an antenatal care facility do not equate to good service provision. Service cannot be delivered properly if the consumer is not aware of its benefits and content.

## Data Availability

The data supporting this research are available from the authors on reasonable request

## References

[1] Islam MM, Masud MS (2018). Determinants of frequency and contents of antenatal care visits in Bangladesh: Assessing the extent of compliance with the WHO recommendations. PLoS One.

[2] Poon LC, Shennan A, Hyett JA (2019). The International Federation of Gynecology and Obstetrics (FIGO) initiative on pre-eclampsia: A pragmatic guide for first-trimester screening and prevention. Int J Gynaecol Obstet.

[3] Muchie KF (2017). Quality of antenatal care services and completion of four or more antenatal care visits in Ethiopia: a finding based on a demographic and health survey. BMC Pregnancy Childbirth.

[4] Agha S, Tappis H (2016). The timing of antenatal care initiation and the content of care in Sindh, Pakistan. BMC Pregnancy Childbirth.

[5] ul Haq Z, Hafeez A, Khanum A, Southall D (2009). Birth preparedness and the role of the private sector: a community survey. J Pak Med Assoc.

[6] Khoso A, Khan AZ, Sayed SA, Rafique G (2016). PERSPECTIVES REGARDING ANTENATAL CARE, DELIVERY AND BREAST FEEDING PRACTICES OF WOMEN FROM BALUCHISTAN, PAKISTAN. J Ayub Med Coll Abbottabad.

[7] Budhwani H, Hearld KR, Harbison H (2015). Individual and Area Level Factors Associated with Prenatal, Delivery, and Postnatal Care in Pakistan. Matern Child Health J.

[8] Shabbir W, Pilz J, Naeem A (2020). A spatial-temporal study for the spread of dengue depending on climate factors in Pakistan (2006-2017). BMC Public Health.

[9] (2016). WHO recommendations on antenatal care for a positive pregnancy experience.

[10] Zakar R, Zakar MZ, Aqil N, Chaudhry A, Nasrullah M (2017). Determinants of maternal health care services utilization in Pakistan: evidence from Pakistan demographic and health survey, 2012-13. J Obstet Gynaecol.

[11] Husain S, Husain S, Izhar R (2019). Women's decision versus couples' decision on using postpartum intrauterine contraceptives. East Mediterr Health J.

[12] (2013). Pakistan Demographic and Health Survey 2012-13.

[13] Byerley BM, Haas DM (2017). A systematic overview of the literature regarding group prenatal care for high-risk pregnant women. BMC Pregnancy Childbirth.

[14] Haddrill R, Jones GL, Mitchell CA, Anumba DO (2014). Understanding delayed access to antenatal care: a qualitative interview study. BMC Pregnancy Childbirth.

[15] Villar J, Ba'aqeel H, Piaggio G (2001). WHO antenatal care randomised trial for the evaluation of a new model of routine antenatal care. Lancet.

[16] Çalışkan Z, Kılıç D, Öztürk S, Atılgan E (2015). Equity in maternal health care service utilization: a systematic review for developing countries. Int J Public Health.

[17] Darling EK, Grenier L, Nussey L, Murray-Davis B, Hutton EK, Vanstone M (2019). Access to midwifery care for people of low socio-economic status: a qualitative descriptive study. BMC Pregnancy Childbirth.

[18] Banke-Thomas OE, Banke-Thomas AO, Ameh CA (2017). Factors influencing utilisation of maternal health services by adolescent mothers in Low-and middle-income countries: a systematic review. BMC Pregnancy Childbirth.

[19] Mason L, Dellicour S, Ter Kuile F (2015). Barriers and facilitators to antenatal and delivery care in western Kenya: a qualitative study. BMC Pregnancy Childbirth.

[20] Warri D, George A (2020). Perceptions of pregnant women of reasons for late initiation of antenatal care: a qualitative interview study. BMC Pregnancy Childbirth.

[21] (2016). United Nations The sustainable development goals report 2016.

[22] Gabrysch S, Nesbitt RC, Schoeps A (2019). Does facility birth reduce maternal and perinatal mortality in Brong Ahafo, Ghana? A secondary analysis using data on 119 244 pregnancies from two cluster-randomised controlled trials. Lancet Glob Health.

[23] Carvajal-Aguirre L, Amouzou A, Mehra V, Ziqi M, Zaka N, Newby H (2017). Gap between contact and content in maternal and newborn care: An analysis of data from 20 countries in sub-Saharan Africa. J Glob Health.

[24] Sharma J, O'Connor M, Rima Jolivet R (2018). Group antenatal care models in low- and middle-income countries: a systematic evidence synthesis. Reprod Health.

[25] Searle AR, Hurley EA, Doumbia SO, Winch PJ (2020). "They Merely Prescribe and I Merely Swallow": Perceptions of Antenatal Pharmaceuticals and Nutritional Supplements Among Pregnant Women in Bamako, Mali. Matern Child Health J.

[26] Jolivet RR, Uttekar BV, O'Connor M, Lakhwani K, Sharma J, Wegner MN (2018). Exploring perceptions of group antenatal Care in Urban India: results of a feasibility study. Reprod Health.

[27] Zaraq Bari M (2019). Child Sexual Abuse and Stolen Dignity: A Socio-Legal Exploration of Child Protection Policies in Pakistan. Pakistan Law Reports.

[28] Kanmaz AG, İnan AH, Beyan E, Ögür S, Budak A (2019). Effect of advanced maternal age on pregnancy outcomes: a single-centre data from a tertiary healthcare hospital. J Obstet Gynaecol.

[29] Javid N, Pu C (2020). Maternal stature, maternal education and child growth in Pakistan: a cross-sectional study. AIMS Public Health.

[30] Tunçalp Ö, Pena-Rosas JP, Lawrie T (2017). WHO recommendations on antenatal care for a positive pregnancy experience-going beyond survival. BJOG.

